# Maternal exposure to fine particulate matter and autism spectrum disorder in children: population based case–control study

**DOI:** 10.3389/fpubh.2026.1848913

**Published:** 2026-06-22

**Authors:** Mengjie Zhang, Yue Zhao, Mengying Zhang, Hongfei Mo, Yan Li

**Affiliations:** 1 The Second Affiliated Hospital of Zhengzhou University, Zhengzhou, Henan, China; 2 The Second Clinical School of Medicine, Zhengzhou University, Zhengzhou, Henan, China; 3 College of Public Health, Zhengzhou University, Zhengzhou, Henan, China; 4 Zhengzhou University, Zhengzhou, Henan, China; 5 School of Basic Medical Sciences, Zhengzhou University, Zhengzhou, Henan, China

**Keywords:** autism spectrum disorder, black carbon, case–control study, maternal exposure, PM_2.5_

## Abstract

**Background:**

Autism spectrum disorder (ASD) is a pervasive neurodevelopmental disorder that can develop in infancy and is caused by a combination of genetic predisposition and environmental factors. Fine particulate matter (PM_2.5_) is one of the most dangerous air pollutants, capable of carrying various toxic substances deep into body tissues and causing complex health effects. Exposure to PM_2.5_ during pregnancy may increase the risk of neurodevelopmental disorders in offspring. This study aimed to investigate the association between maternal exposure to PM_2.5_ and its major components during pregnancy and childhood ASD, and to identify critical gestational windows of vulnerability.

**Methods:**

All participants were recruited exclusively from the Xinyang Central Hospital, and covariates were ascertained through structured clinical interviews. PM_2.5_ and its components, including sulfate ( ${\mathrm{SO}}_{4}^{2-}$ ), nitrate ( ${\mathrm{NO}}_{3}^{-}$ ), ammonium ( ${\mathrm{NH}}_{4}^{+}$ ), organic matter (OM), and black carbon (BC), were derived from Tracking Air Pollution in China (TAP). Statistical analysis was performed using SPSS, employing the chi-square test, rank-sum test, and binary logistic regression analysis.

**Results:**

Positive associations were observed between PM_2.5_ and ASD (OR = 5.197, 95% CI: 3.294–8.200), and between ${\mathrm{SO}}_{4}^{2-}$ , ${\mathrm{NO}}_{3}^{-}$ , ${\mathrm{NH}}_{4}^{+}$ , BC, and OM and ASD. Monthly risk estimates for all pollutants exhibited a right-skewed distribution, with the strongest associations consistently observed during the gestational months 4–7.

**Conclusion:**

Maternal exposure to PM_2.5_, ${\mathrm{SO}}_{4}^{2-}$ , ${\mathrm{NO}}_{3}^{-}$ , ${\mathrm{NH}}_{4}^{+}$ , BC, and OM was associated with ASD in children, particularly ${\mathrm{SO}}_{4}^{2-}$ and BC. The 4th–7th gestational months represent a critical window of vulnerability. These findings support targeted public health interventions to reduce prenatal air pollution exposure.

## 1 Introduction

Autism spectrum disorder (ASD) is a neurodevelopmental disorder characterized by impaired social interaction and communication skills and restricted and repetitive behaviors. ASD has a high prevalence and serious impact, with far-reaching implications for individuals, families, and society. Currently, there are few effective treatments available, making ASD a significant public health burden (1, 2).

Fine particulate matter (PM_2.5_) is an air pollutant emitted primarily from industrial processes, vehicle exhaust, and biomass combustion. Recent evidence suggests that exposure to PM_2.5_ has been associated with an increased risk of mental health outcomes such as depression, anxiety, and cognitive decline (3). PM_2.5_ contains various components including sulfate ( ${\mathrm{SO}}_{4}^{2-}$ ), nitrate ( ${\mathrm{NO}}_{3}^{-}$ ), ammonium ( ${\mathrm{NH}}_{4}^{+}$ ), organic matter (OM), and black carbon (BC) (4, 5), which may exert different effects on ASD. ${\mathrm{SO}}_{4}^{2-}$ is a sulfur by-product produced during fossil fuel combustion. Studies have shown that high levels of ${\mathrm{SO}}_{4}^{2-}$ cause oxidative stress and neuroinflammation, potentially contributing to ASD (6). ${\mathrm{NO}}_{3}^{-}$ , emitted primarily from automobile and power plant combustion, also contributes to oxidative stress and inflammation (7). ${\mathrm{NH}}_{4}^{+}$ formed through the reaction of ammonia with other substances in the air, remains a common component of PM_2.5_ and has been linked to neurodevelopmental outcomes. Research suggests that maternal and early postnatal exposure to ${\mathrm{NH}}_{4}^{+}$ may have deleterious effects on brain development (8). OM consists of a variety of substances, including volatile organic compounds (VOCs) and polycyclic aromatic hydrocarbons (PAHs), which can adversely affect fetal neurodevelopment (9) and may increase the risk of ASD. BC, a byproduct from combustion sources such as automobile exhaust and biomass burning, is also a concern. However, more studies are needed to determine the specific association between BC exposure and ASD. Overall, more research is needed to understand the relationship between various components of PM_2.5_ and ASD and to develop effective strategies to minimize the adverse health effects of air pollution.

Maternal PM_2.5_ exposure has emerged as a plausible environmental risk factor for ASD. However, the majority of previous studies have focused on average gestational exposure rather than identifying critical windows of vulnerability. Additionally, the specific contributions of individual PM_2.5_ components remain poorly understood. Clarifying these associations and timing-specific risks is essential for designing effective preventive interventions. This study employed a population-based case–control design to examine the associations between maternal exposure to PM_2.5_ and its components during pregnancy and the risk of childhood ASD. It further characterized the monthly exposure–response relationships throughout gestation to identify critical windows of susceptibility. Findings from this observational analysis may inform future mechanistic investigations and public health policies aimed at reducing prenatal air pollution-related neurodevelopmental risks.

## 2 Materials and methods

### 2.1 Participants

**Figure 1:**
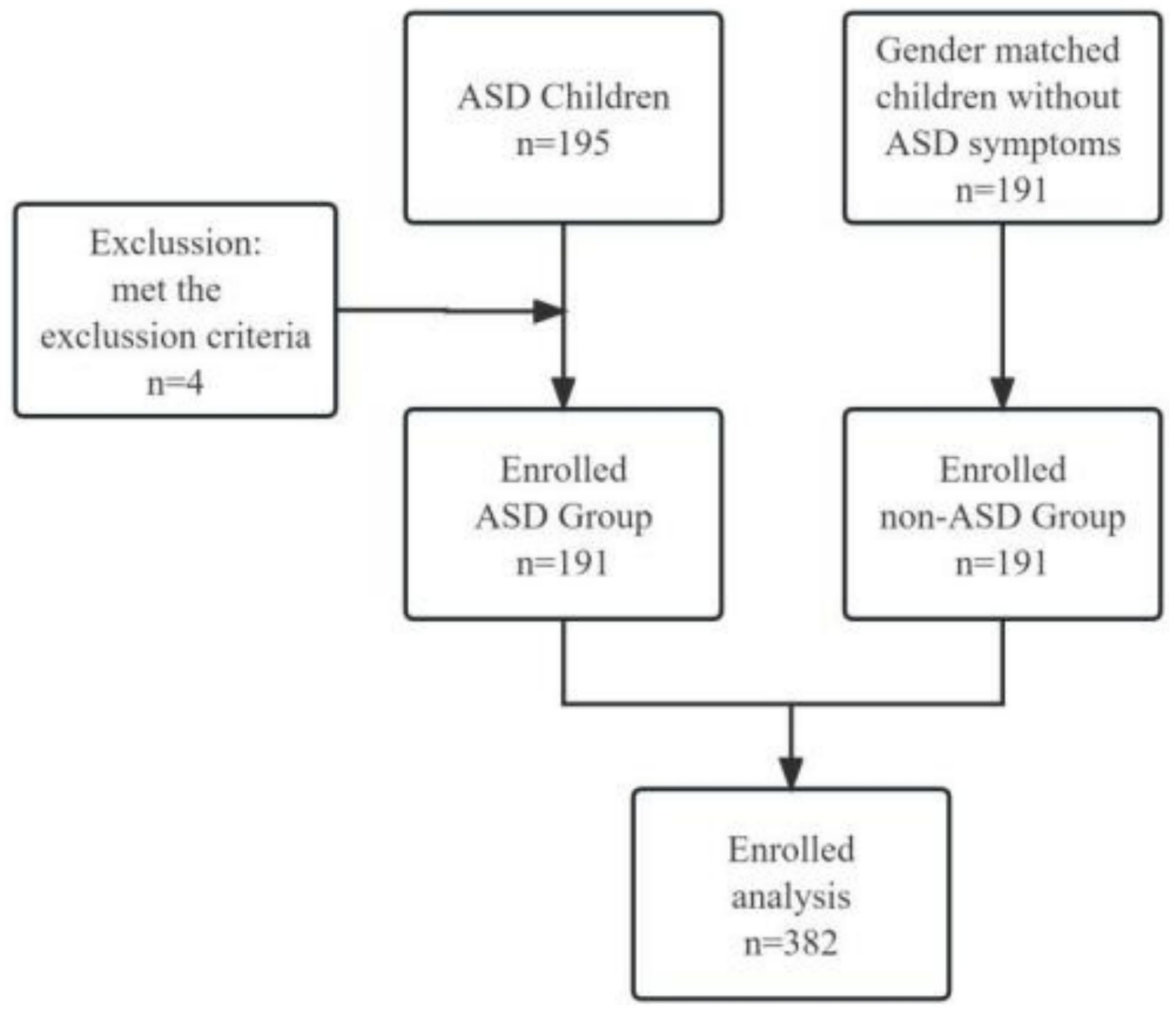
Flow chart depicting subject selection.

All subjects were recruited from Xinyang Central Hospital from 1 May 2020 to 31 December 2022 and were divided into two groups: ASD group (case group) and non-ASD group (control group). Participants were selected based on inclusion and exclusion criteria (see Figure 1). A total of 382 participants (306 boys and 76 girls) were included, with ASD patients (*n* = 191) as the case group and non-ASD children (*n* = 191) as the control group. The mean age was 6.16 ± 2.95 years.

Inclusion criteria: ① Age: Range from 2 to 12 years, ensuring participants were within a critical neurodevelopmental period; ② Case group: Children with a confirmed diagnosis of ASD by an experienced clinician using the Diagnostic and Statistical Manual of Mental Disorders Fifth Edition (DSM-5) criteria; ③ Control group: Children evaluated in the healthcare department who do not have symptoms of ASD; ④ Residence: Mothers resided permanently in Xinyang City, Henan Province during pregnancy, ensuring consistent exposure to similar environmental pollutants during the fetal period; and ⑤ Informed consent: Legal guardians provided signed informed consent.

Exclusion criteria: ① Comorbidities: Severe intellectual disability, genetic syndromes associated with ASD, and neurological disorders with overlapping symptoms to ASD; ② Drug therapy: Participants presently receiving medications that may affect neurodevelopment, including antipsychotics or antiepileptics; ③ Pre-existing health conditions: Children with congenital and chronic diseases, severe head trauma, and central nervous system surgery; ④ Language or communication limitations: Participants with severe language or communication impairments that may affect their ability to provide reliable data or fully participate in the assessment.

The study was approved by the Life Science Ethics Review Committee of Zhengzhou University (No. ZZUIRB2023-256).

### 2.2 Demographic information

In this study, demographic information was collected from participants at the time of admission, including gender, age, birth weight, height, weight, anamnesis, allergies, vaccination history, health status, delivery method, and premature status of children, along with the pregnancy history and miscarriage history of participants’ mothers. Body mass index (BMI) was calculated as weight divided by the square of height (kg/m^2^). All participants were vaccinated. These data were obtained through on-site interviews conducted by trained, experienced clinicians with participants’ mothers.

### 2.3 Fine particulate matter exposure assessments

Data on PM_2.5_ exposure for this study were obtained by matching the latitude and longitude coordinates of the residences of the participants’ mothers during pregnancy with PM_2.5_ pollution data at a 1 km resolution. This residence information was provided by the participants’ legal guardians, and was converted from written descriptions to latitude and longitude coordinates. The 1 km resolution PM_2.5_ pollution data were obtained from Tracking Air Pollution in China (TAP) (5).

TAP constructed a two-level machine learning model to generate complete day-by-day coverage of PM_2.5_ concentrations (μg/m^3^). In the first-level model, a high-pollution event index was defined based on the observed data, and the training dataset was resampled using the synthetic minority over-sampling technique (SMOTE) to increase the proportion of high-pollution events. Based on the resampled training dataset and the random forest algorithm (RFA), high-pollution events were predicted. In the second-level model, the residuals between PM_2.5_ concentration simulated by the community multiscale air quality (CMAQ) and the observed PM_2.5_ concentrations were used to build a second RFA model (10–12). This two-level machine learning model initially produced a 10 km resolution daily average PM_2.5_ dataset.

From the 10 km dataset, TAP further established the PM_2.5_ component concentration dataset, including ${\mathrm{SO}}_{4}^{2-}$ , ${\mathrm{NO}}_{3}^{-}$ , ${\mathrm{NH}}_{4}^{+}$ , OM, and BC. Component information of PM_2.5_ was obtained from operational CMAQ simulations, constrained by total PM_2.5_ concentration. To correct the CMAQ model simulation bias, the dust emission simulation module was improved, and a correction model was developed using the observed PM_2.5_ component concentration data and the extreme gradient boosting (XGBoost) algorithm. This yielded more accurate data between the PM_2.5_ and its component conversion factors (13, 14). TAP also built a machine learning model by fusing high-resolution satellite remote sensing aerosol optical depth (AOD) data with environmental spatial data (such as road networks). This model is used to estimate PM_2.5_ concentrations and its components, providing complete day-by-day coverage at a 1 km resolution. To accurately characterize the spatial characteristics data closely related to pollutant emissions and their temporal variations, a continuous year-by-year environmental spatial dataset, including population distribution, road network, vegetation cover, surface type, and elevation, was first constructed using a geographic information model and a statistical model to integrate statistical data with gridded public data. Finally, an RFA model was built using the residuals between the 10 km resolution PM_2.5_ and its component concentration and their observed concentration. This approach enhanced the response of each parameter to the changes in concentration. As a result, data regarding 1 km resolution PM_2.5_ and its component concentration were obtained, ensuring complete spatial and temporal coverage (15).

In this study, data on 1 km resolution PM_2.5_ and its component concentration data were extracted from the collected datasets that fully covered Henan Province. These data were matched with the latitude and longitude coordinates of residences of participants’ mothers during pregnancy to filter and calculate the monthly average exposure levels to PM_2.5_, ${\mathrm{SO}}_{4}^{2-}$ , ${\mathrm{NO}}_{3}^{-}$ , ${\mathrm{NH}}_{4}^{+}$ , BC, and OM from month 0 to month 9 of pregnancy (or from month 7 or 8 for premature participants).

### 2.4 Cut-off point selection

According to the World Health Organization (WHO) air quality guidelines, the recommended annual average concentration of PM_2.5_ should not exceed 10 μg/m^3^. In addition, the guidelines also limits the 24-h average concentration to 25 μg/m^3^ (4). Additionally, the United States Environmental Protection Agency (US EPA) set relatively lenient acceptable standards of 12 μg/m^3^ for the annual average and 35 μg/m^3^ for the 24-h average (16). According to the ambient air quality standards (GB 3095–2012) issued by the Ministry of Ecology and Environment of the People’s Republic of China (MEEPRC), the annual average and 24-h average PM_2.5_ concentrations are limited to 35 μg/m^3^ and 75 μg/m^3^, respectively (17). However, none of these criteria apply to this study. First, the important factor in this study is the monthly average exposure level, and there are no existing guidelines to inform its categorization. The annual and daily average guidelines are also not directly usable. Second, the minimum annual average exposure among all participants was 38.78 μg/m^3^, which exceeded the maximum exposure standard set by the MEEPRC, rendering the absence of any guidelines for this study. Furthermore, BC and OM in the air are not directly regulated by any specific standards, including those developed by the WHO, US EPA, and MEEPRC. No clinical or regulatory guidelines presently define exposure thresholds for prenatal PM_2.5_ in relation to neurodevelopmental risk, including ASD. To enable balanced group comparisons and stable risk estimation across all gestational months, which is essential for identifying critical windows of vulnerability, this study dichotomized exposure using the sample mean. This approach maximizes statistical power and is widely used in environmental epidemiology when no established cut-offs are available. In fact, using the mean as a cut-off point has been shown to be feasible in previous studies, particularly those conducted in highly polluted areas (18, 19).

### 2.5 Data processing and statistical analysis

The initial processing of the data was divided into three parts. The first part is the preprocessing of demographic data: ① Data were entered using EpiData 3.1, and the original data were organized in Microsoft Excel 2010; ② The written description of the participants’ mothers’ residences during pregnancy was converted into latitude and longitude coordinates; ③ The “EDATE” function in Excel was used to extrapolate the date of birth of the participants’ mothers from month 0 to month 9 of pregnancy. The second part is combining demographic data with PM_2.5_ pollution data: ① Excel was used to integrate all 1 km resolution PM_2.5_ daily average data from 2006 to 2022 into a single data table; ② The datasets provided by TAP included PM_2.5_ and its component concentration data with 1 km resolution, latitude and longitude coordinates, and date. The “XLOOKUP” function in Excel was used to filter the PM_2.5_ and its components daily average exposure data from the pollution data table for month 0 to month 9 of pregnancy for the participants’ mothers, using the date and latitude/longitude coordinates as matching conditions. The third part involved establishing the database. The monthly average exposure data for the participants’ mothers were calculated from month 0 to month 9 of pregnancy using the “AVERAGE” function in Excel, and the values were assigned to build the final database.

Chi-square and rank-sum tests were performed on the demographic data. Binary logistic regression analysis was conducted to estimate the association between PM_2.5_ and its components exposures with ASD, and the statistically significant demographic variables were screened as covariates. Forest plots illustrating the association of each pollutant with ASD were generated using GraphPad Prism 8, to visualize the degree of associations and their trends. All data analyses were conducted using SPSS 28.0, and *p*-values of <0.05 were considered to be statistically significant.

## 3 Results

### 3.1 Demographic characteristics

A total of 382 participants were included in this study, with ASD patients (*n* = 191) as the case group and non-ASD children (*n* = 191) as the control group; 306 boys (80.1%) and 76 girls (19.9%). The average age was 6.16 ± 2.95 years. The difference between the two groups of participants in age, BMI, and mother’s pregnancy history were statistically significant (all *p* < 0.001). There was no statistically significant difference between the two groups in birth weight (*p* = 0.992), anamnesis (*p* = 0.704), allergy (*p* = 0.358), health status (*p* = 0.947), delivery method (*p* = 0.411), premature (*p* = 0.792), and mother’s miscarriage history (*p* = 0.190) (see Table 1).

**Table 1:** Demographic characteristics of ASD group and non-ASD group.

Characteristics, *n*%	Sample capacity	ASD group	Non-ASD group	T	*p*-value
	*N* = 382	*n* = 191 (50.00)	*n* = 191 (50.00)		
Gender					
Boys	306(80.10)	153(80.10)	153(80.10)		
Girls	76(19.90)	38(19.90)	38(19.90)		
Age	6.16 ± 2.95	4.52 ± 1.77	7.81 ± 2.96	−11.203^a^	<0.001
Birth weight	3.40 ± 0.52	3.41 ± 0.51	3.40 ± 0.54	−0.010^a^	0.992
BMI	15.63 ± 3.45	15.23 ± 2.53	16.03 ± 4.13	−10.073^a^	<0.001
Anamnesis				0.145^b^	0.704
Yes	30(7.85)	14(7.33)	16(8.38)		
No	352(92.15)	177(92.67)	175(91.62)		
Allergy				0.844^b^	0.358
Yes	20(5.24)	12(6.28)	8(4.19)		
No	362(94.76)	179(93.72)	183(95.81)		
Health status				0.001^b^	0.947
Poor	2(0.52)	2(1.05)	0(0.00)		
Fine	345(90.31)	167(87.43)	178(93.19)		
Good	35(9.16)	22(11.52)	13(6.81)		
Delivery method				0.677^b^	0.411
Eutocia	172(45.03)	82(42.93)	90(47.12)		
Cesarean	210(54.97)	109(57.07)	101(52.88)		
Premature				0.069^b^	0.792
Yes	15(3.93)	8(4.19)	7(3.66)		
No	367(96.07)	183(95.81)	184(96.34)		
Mother’s pregnancy history				17.941^b^	<0.001
Yes	127(33.25)	83(43.46)	44(23.04)		
No	255(66.75)	108(56.54)	147(76.96)		
Mother’s miscarriage History				1.720^b^	0.190
Yes	31(8.12)	19(9.95)	12(6.28)		
No	351(91.88)	172(90.05)	179(93.72)		

^a^ Rank-sum test; ^b^ chi-square test.

### 3.2 Association between mean maternal PM_2.5_ exposure and ASD

In the first subgroup, the independent variables were classified as dichotomous (high vs. low exposure). Before adjusting for covariates, the association of PM_2.5_ with ASD was 5.182, (3.347–8.023); the association of ${\mathrm{SO}}_{4}^{2-}$ , ${\mathrm{NO}}_{3}^{-}$ , ${\mathrm{NH}}_{4}^{+}$ , BC, and OM with ASD were 7.318 (4.622–11.585), 3.938 (2.575–6.024), 7.203 (4.583–11.321), 3.506 (2.301–5.342) and 10.712 (6.597–17.393), respectively. After adjusting for covariates, the association of PM_2.5_ with ASD was 5.197 (3.294–8.200); the association of ${\mathrm{SO}}_{4}^{2-}$ , ${\mathrm{NO}}_{3}^{-}$ , ${\mathrm{NH}}_{4}^{+}$ , BC, and OM with ASD were 7.215 (4.475–11.631), 3.950 (2.544–6.134), 7.218 (4.504–11.567), 3.509 (2.270–5.426), and 10.912 (6.550–18.181), respectively. All *p*-values of <0.001 were considered statistically significant.

In the second subgroup, the independent variables were classified as continuous variables (per μg/m^3^ increase). Before adjusting for covariates, the association of PM_2.5_ with ASD was 1.110 (1.083–1.137); the association of ${\mathrm{SO}}_{4}^{2-}$ , ${\mathrm{NO}}_{3}^{-}$ , ${\mathrm{NH}}_{4}^{+}$ , BC, and OM with ASD were 1.735 (1.538–1.957), 1.483 (1.347–1.632), 1.829 (1.595–2.098), 1.521 (1.370–1.689), and 12.385 (7.048–21.763), respectively. After adjusted for covariates, the association of PM_2.5_ with ASD was 1.112 (1.083–1.138); the association of ${\mathrm{SO}}_{4}^{2-}$ , ${\mathrm{NO}}_{3}^{-}$ , ${\mathrm{NH}}_{4}^{+}$ , BC, and OM with ASD were 1.738 (1.533–1.969), 1.489 (1.349–1.644), 1.829 (1.588–2.106), 1.528 (1.371–1.704), and 12.852 (7.161–23.068), respectively. All *p* values of <0.001 were considered statistically significant (see Table 2).

**Table 2:** Weighted association of mean pregnancy PM_2.5_ and its components exposures with ASD.

Exposure substance	Dichotomous (high vs. low exposure)		Continuous (per 1 μg/m^3^ increase)	
	OR (95% CI)	*P*-value	OR (95% CI)	*p*-value
PM_2.5_				
Model I	5.18 (3.35–8.02)	<0.001	1.11 (1.08–1.14)	<0.001
Model II	5.20 (3.29–8.20)	<0.001	1.11 (1.08–1.14)	<0.001
${\mathrm{SO}}_{4}^{2-}$				
Model I	7.32 (4.62–11.59)	<0.001	1.74 (1.54–1.96)	<0.001
Model II	7.22 (4.48–11.63)	<0.001	1.74 (1.53–1.97)	<0.001
${\mathrm{NO}}_{3}^{-}$				
Model I	3.94 (2.58–6.02)	<0.001	1.48 (1.35–1.63)	<0.001
Model II	3.95 (2.54–6.13)	<0.001	1.49 (1.35–1.64)	<0.001
${\mathrm{NH}}_{4}^{+}$				
Model I	7.20 (4.58–11.32)	<0.001	1.84 (1.60–2.10)	<0.001
Model II	7.22 (4.50–11.57)	<0.001	1.83 (1.59–2.11)	<0.001
OM				
Model I	3.51 (2.30–5.34)	<0.001	1.52 (1.37–1.69)	<0.001
Model II	3.51 (2.27–5.43)	<0.001	1.53 (1.37–1.70)	<0.001
BC				
Model I	10.71 (6.60–17.39)	<0.001	12.39 (7.05–21.76)	<0.001
Model II	10.91 (6.55–18.18)	<0.001	12.85 (7.16–23.07)	<0.001

Model I, original model; Model II, excluded statistically significant covariates, same as below.

### 3.3 Trends in the association between monthly mean maternal PM_2.5_ exposure and ASD

In the first subgroup, the independent variables were classified as dichotomous (high vs. low exposure). After adjusted for covariates, the OR of monthly mean PM_2.5_ with ASD were 0.827, 1.419, 1.540, 1.778, 2.287, 2.644, 2.537, 2.482, 1.746, and 1.166, respectively, from month 0 to month 9 of pregnancy, among which month 2 to month 8 were statistically significant (*p* < 0.05); the OR of monthly mean ${\mathrm{SO}}_{4}^{2-}$ with ASD were 1.320, 2.118, 2.533, 2.712, 2.893, 3.814, 3.918, 3.286, 2.693, and 1.493, respectively, among which month 1 to month 9 were statistically significant; the OR of monthly mean ${\mathrm{NO}}_{3}^{-}$ with ASD were 0.653, 0.941, 1.104, 1.385, 1.836, 2.357, 2.300, 2.321, 1.506, and 1.031, respectively, among which month 4 to month 7 were statistically significant; the OR of monthly mean ${\mathrm{NH}}_{4}^{+}$ with ASD were 0.901, 1.364, 1.665, 1.791, 2.360, 2.804, 2.548, 2.945, 1.366, and 1.212, respectively, among which month 2 to month 7 were statistically significant; the OR of monthly mean OM with ASD were 0.775, 0.701, 1.101, 1.388, 1.799, 2.397, 2.521, 2.291, 1.403, and 1.046, respectively, among which month 3 to month 7 were statistically significant; the OR of monthly mean BC with ASD were 1.060, 1.512, 2.002, 2.289, 2.288, 3.316, 3.914, 3.551, 2.231, and 1.297, respectively, among which month 2 to month 8 were statistically significant.

In the second subgroup, the independent variables were classified as continuous variables (per 1 μg/m^3^ increase). After adjusted for covariates, the OR of monthly mean PM_2.5_ with ASD were 1.003, 1.008, 1.015, 1.016, 1.019, 1.025, 1.028, 1.025, 1.017, and 1.007, respectively, from month 0 to month 9 of pregnancy, among which month 2 to month 9 were statistically significant; the OR of monthly mean ${\mathrm{SO}}_{4}^{2-}$ with ASD were 1.052, 1.096, 1.147, 1.148, 1.169, 1.210, 1.231, 1.191, 1.125, and 1.062, respectively, all *p* values of <0.05 were considered statistically significant; the OR of monthly mean ${\mathrm{NO}}_{3}^{-}$ with ASD were 0.995, 1.013, 1.039, 1.042, 1.056, 1.074, 1.088, 1.077, 1.045, and 1.016, respectively, among which month 2 to month 8 were statistically significant; the OR of monthly mean ${\mathrm{NH}}_{4}^{+}$ with ASD were 1.015, 1.047, 1.096, 1.099, 1.119, 1.158, 1.181, 1.160, 1.106, and 1.047, respectively, among which month 2 to month 9 were statistically significant; the OR of monthly mean OM with ASD were 0.994, 1.014, 1.044, 1.049, 1.060, 1.085, 1.102, 1.090, 1.059, and 1.019, respectively, among which month 2 to month 8 were statistically significant; the OR of monthly mean BC with ASD were 1.140, 1.264, 1.556, 1.576, 1.606, 1.855, 2.035, 1.952, 1.588, and 1.238, respectively, among which month 1 to month 9 were statistically significant (see Table 3).

**Table 3:** Weighted association of monthly mean pregnancy PM_2.5_ exposure and ASD.

Exposed substance	Pregnancy month	Dichotomous (high vs. low exposure)		Continuous (per 1 μg/m^3^ increase)	
		OR(95% CI)	*p*-value	OR (95% CI)	*p*-value
PM_2.5_	Month 0	0.83 (0.58–1.33)	0.526	1.00 (1.00–1.01)	0.510
	Month 1	1.42 (0.93–2.16)	0.103	1.01(1.00–1.02)	0.058
	Month 2	1.54 (1.01–2.34)	0.043	1.02 (1.01–1.02)	<0.001
	Month 3	1.78 (1.16–2.72)	0.008	1.02 (1.01–1.02)	<0.001
	Month 4	2.29 (1.49–3.51)	<0.001	1.02(1.01–1.03)	<0.001
	Month 5	2.64 (1.71–4.09)	<0.001	1.03 (1.02–1.04)	<0.001
	Month 6	2.54 (1.57–3.60)	<0.001	1.03 (1.02–1.04)	<0.001
	Month 7	2.48 (1.61–3.83)	<0.001	1.03 (1.02–1.04)	<0.001
	Month 8	1.75 (1.15–2.66)	0.009	1.02 (1.01–1.03)	<0.001
	Month 9	1.17 (0.80–1.69)	0.418	1.01 (1.00–1.01)	0.043
${\mathrm{SO}}_{4}^{2-}$	Month 0	1.32 (0.87–2.01)	0.195	1.05 (1.00–1.10)	0.040
	Month 1	2.12 (1.39–3.24)	0.001	1.10 (1.04–1.15)	0.001
	Month 2	2.53 (1.66–3.88)	<0.001	1.15 (1.09–1.21)	<0.001
	Month 3	2.71 (1.77–4.16)	<0.001	1.15 (1.09–1.21)	<0.001
	Month 4	2.89 (1.88–4.45)	<0.001	1.17 (1.11–1.24)	<0.001
	Month 5	3.81 (2.45–5.94)	<0.001	1.21 (1.14–1.28)	<0.001
	Month 6	3.92 (2.52–6.10)	<0.001	1.23 (1.16–1.31)	<0.001
	Month 7	3.29 (2.11–5.12)	<0.001	1.19 (1.13–1.26)	<0.001
	Month 8	2.69 (1.76–4.12)	<0.001	1.13 (1.07–1.18)	<0.001
	Month 9	1.49 (1.13–2.17)	0.035	1.06 (1.02–1.11)	0.006
${\mathrm{NO}}_{3}^{-}$	Month 0	0.65 (0.43–1.00)	0.049	1.00 (0.97–1.02)	0.707
	Month 1	0.94 (0.62–1.43)	0.773	1.01 (0.99–1.04)	0.336
	Month 2	1.10 (0.73–1.68)	0.641	1.04 (1.01–1.07)	0.008
	Month 3	1.39 (0.91–2.11)	0.129	1.04 (1.01–1.07)	0.003
	Month 4	1.84 (1.20–2.82)	0.005	1.06 (1.03–1.09)	<0.001
	Month 5	2.36 (1.52–3.65)	<0.001	1.07 (1.04–1.11)	<0.001
	Month 6	2.30 (1.49–3.55)	<0.001	1.09 (1.05–1.12)	<0.001
	Month 7	2.32 (1.50–3.59)	<0.001	1.08 (1.05–1.11)	<0.001
	Month 8	1.51 (0.99–2.29)	0.056	1.05 (1.02–1.07)	0.001
	Month 9	1.03 (0.71–1.50)	0.872	1.02 (0.99–1.04)	0.195
${\mathrm{NH}}_{4}^{+}$	Month 0	0.90 (0.59–1.37)	0.628	1.02 (0.97–1.06)	0.505
	Month 1	1.36 (0.90–2.07)	0.147	1.05 (1.00–1.10)	0.051
	Month 2	1.67 (1.10–2.53)	0.017	1.10 (1.04–1.15)	<0.001
	Month 3	1.79 (1.17–2.74)	0.007	1.10 (1.05–1.15)	<0.001
	Month 4	2.36 (1.54–3.63)	<0.001	1.12 (1.07–1.18)	<0.001
	Month 5	2.80 (1.81–4.34)	<0.001	1.16 (1.10–1.22)	<0.001
	Month 6	2.55 (1.65–3.93)	<0.001	1.18 (1.12–1.25)	<0.001
	Month 7	2.95 (1.90–4.56)	<0.001	1.16 (1.10–1.22)	<0.001
	Month 8	1.37 (0.85–2.19)	0.197	1.11 (1.06–1.16)	<0.001
	Month 9	1.21 (0.84–1.76)	0.310	1.05 (1.01–1.09)	0.029
OM	Month 0	0.78 (0.51–1.19)	0.240	0.99 (0.97–1.02)	0.701
	Month 1	0.70 (0.46–1.05)	0.097	1.01 (0.98–1.05)	0.382
	Month 2	1 (0.73–1.67)	0.650	1.04 (1.01–1.08)	0.011
	Month 3	1.39 (0.91–2.11)	0.126	1.05 (1.02–1.08)	0.003
	Month 4	1.80 (1.18–2.75)	0.007	1.06 (1.03–1.10)	<0.001
	Month 5	2.40 (1.55–3.71)	<0.001	1.09 (1.05–1.11)	<0.001
	Month 6	2.52 (1.63–3.90)	<0.001	1.10 (1.06–1.14)	<0.001
	Month 7	2.29 (1.49–3.54)	<0.001	1.09 (1.05–1.13)	<0.001
	Month 8	1.40 (0.92–2.14)	0.115	1.06 (1.03–1.09)	<0.001
	Month 9	1.05 (0.72–1.52)	0.814	1.02 (0.99–1.05)	0.196
BC	Month 0	1.06 (0.70–1.62)	0.785	1.14 (0.96–1.35)	0.136
	Month 1	1.51 (0.99–2.31)	0.056	1.26 (1.05–1.52)	0.014
	Month 2	2.00 (1.31–3.05)	0.001	1.56 (1.27–1.91)	<0.001
	Month 3	2.29 (1.49–3.51)	<0.001	1.58 (1.29–1.92)	<0.001
	Month 4	2.29 (1.49–3.51)	<0.001	1.61 (1.32–1.96)	<0.001
	Month 5	3.32 (2.13–5.16)	<0.001	1.86 (1.50–2.30)	<0.001
	Month 6	3.91 (2.50–6.14)	<0.001	2.04 (1.62–2.56)	<0.001
	Month 7	3.55 (2.27–5.56)	<0.001	1.95 (1.56–2.45)	<0.001
	Month 8	2.23 (1.46–3.41)	<0.001	1.59 (1.32–1.92)	<0.001
	Month 9	1.30 (0.89–1.89)	0.173	1.24 (1.05–1.46)	0.011

**Figure 2:**
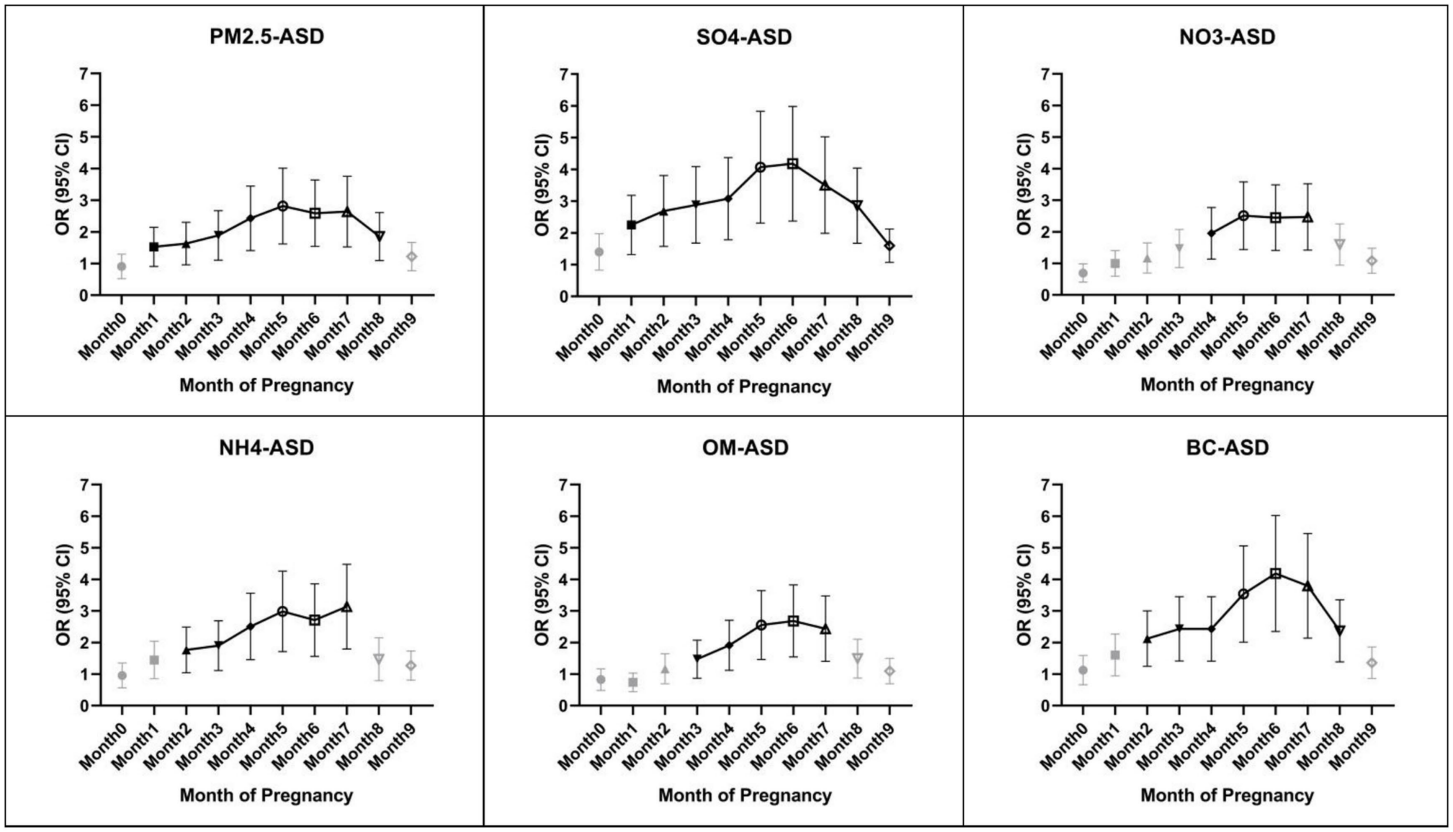
Graphs depicting trends in the association between monthly mean pregnancy PM_2.5_ exposure and ASD.

Six forest plots were generated based on the statistical results of the first subgroup, where ORs that were not statistically significant were marked in gray and those that are statistically significant were marked in black and connected by lines. The *x*-axis represents the month of pregnancy, ranging from month 0 to month 9, and the *y*-axis represents the OR (95% CI). The forest plots show that in the association between pregnancy PM_2.5_ exposure and ASD, the association showed an increasing trend from month 1 to month 4, and higher levels from month 4 to month 7, and declined after month 7, forming an overall right skewed distribution. In the association between pregnancy ${\mathrm{SO}}_{4}^{2-}$ exposure and ASD, the association showed an increasing trend from month 1 to month 5, a decreasing trend from month 6 to month 9, and a higher level from month 4 to month 8, also showing an overall right skewed distribution. In the association between pregnancy ${\mathrm{NO}}_{3}^{-}$ exposure and ASD, the association was statistically significant from month 4 to month 7, and the level of association approximated. In the association between pregnancy ${\mathrm{NH}}_{4}^{+}$ exposure and ASD, the association showed an overall increasing trend from month 2 to month 7, with a higher level from month 5 to month 7. In the association between pregnancy OM exposure and ASD, the association showed an overall increasing trend from month 3 to month 7, with a higher level from month 5 to month 7. In the association between pregnancy BC exposure and ASD, the association showed an increasing trend from month 2 to month 6, a decreasing trend from month 6 to month 8, and a higher level from month 5 to month 7, with an overall right skewed distribution. Overall, exposure to components of PM_2.5_ such as ${\mathrm{SO}}_{4}^{2-}$ , ${\mathrm{NO}}_{3}^{-}$ , ${\mathrm{NH}}_{4}^{+}$ , BC, and OM during pregnancy was associated with ASD risk to varying degrees, with ${\mathrm{SO}}_{4}^{2-}$ and BC demonstrating the strongest effects (see Figure 2).

## 4 Discussion

This study found a significant association between exposure to PM_2.5_ and its components ${\mathrm{SO}}_{4}^{2-}$ , ${\mathrm{NO}}_{3}^{-}$ , ${\mathrm{NH}}_{4}^{+}$ , BC, and OM during pregnancy and childhood ASD. The forest plots showed that among PM_2.5_ and its components, the average OR of monthly exposure during pregnancy showed an approximate right skewed distribution characteristic, with all pollutants showing higher levels of association from month 4 to month 7. While prior epidemiological studies have confirmed associations between ${\mathrm{SO}}_{4}^{2-}$ , ${\mathrm{NO}}_{3}^{-}$ exposure during pregnancy and ASD in children (20, 21), the present discussion primarily focuses on ${\mathrm{NH}}_{4}^{+}$ , OM, and BC.

The study results confirmed a robust association between prenatal ${\mathrm{NH}}_{4}^{+}$ exposure and elevated ASD risk in offspring. To further understand this result, two reasons were considered. Firstly, ${\mathrm{NH}}_{4}^{+}$ exposure could directly affect biochemical processes in the body, which may lead to neurodevelopmental abnormalities. An animal study demonstrated that disturbances in gene expression in the brains of mice exposed to elevated levels of ${\mathrm{NH}}_{4}^{+}$ , particularly genes involved in neuronal development and synaptic plasticity (22). In addition, another study showed that exposure to air pollutants during pregnancy was associated with an increase in markers of oxidative stress in maternal and cord blood, suggesting a link between airborne ${\mathrm{NH}}_{4}^{+}$ exposure and altered oxidative stress responses (23). Given that oxidative stress is implicated in the pathogenesis of ASD, it is plausible that ${\mathrm{NH}}_{4}^{+}$ exposure contributes to ASD risk through dysregulation of oxidative stress processes. Secondly, the genetic factors may influence susceptibility to ${\mathrm{NH}}_{4}^{+}$ -induced ASD risk. Individual genetic variation plays a key role in determining individual susceptibility to environmental risk factors. Recent genome-wide association studies have identified multiple genes associated with ASD, many of which are involved in various aspects of neuronal development and function, such as synaptic pruning, axonal growth, and neurotransmission. However, the interaction between genetic susceptibility and environmental factors (particularly ${\mathrm{NH}}_{4}^{+}$ exposure) remain understudied. A previous study demonstrated that maternal exposure to air pollution altered common ASD-associated genes, with genetic variation linked to cognitive function (24). This suggests that genetic susceptibility combined with exposure to airborne ${\mathrm{NH}}_{4}^{+}$ may contribute to ASD risk and severity. Future studies should focus on identifying specific genetic variants that increase the risk of ${\mathrm{NH}}_{4}^{+}$ -associated ASD.

The results showed a significant association between pregnancy OM exposure and ASD. The emergence of this result is due to both the biochemical mechanisms and genetic factors. Firstly, airborne OM consists of a complex mixture of compounds, including PAHs and VOCs, these substances have the potential to cross the placental barrier and negatively impact fetal development. A study showed that maternal exposure to PAHs was associated with altered DNA methylation patterns, which may disrupt gene expression patterns critical for neurodevelopment (25). Another study found that exposure to PAHs during pregnancy was associated with DNA methylation changes in genes related to neuronal development and function (26). These findings suggest that OM exposure during pregnancy may influence neurodevelopment by modifying DNA methylation patterns, potentially contributing to the development of ASD. Secondly, recent advancements in genetic research have also identified immune-regulation-associated genes in ASD, and the interaction between OM exposure and genetic variations may exacerbate the risk of ASD. There was study demonstrating that specific genetic variants related to immune dysregulation significantly modified the relationship between maternal exposure to airborne OM pollution and ASD risk (27). This suggests that individuals with certain genetic variations may have an increased susceptibility to the detrimental effects of OM exposure, increasing ASD risk. Further investigations are needed to identify additional genetic factors influencing the association between OM exposure and ASD risk.

A significant association was also found between pregnancy BC exposure and ASD, and which may be analyzed from both biochemical and genetic perspectives. BC generated through incomplete combustion of fossil fuels, carries toxic organic compounds and heavy metals that can induce oxidative stress and inflammation. Oxidative stress occurs when there is an imbalance between the production of reactive oxygen species (ROS) and the antioxidant defense system, which could lead to cellular damage and dysfunction. A review demonstrated that BC exposure could increase oxidative stress in the placenta, potentially affecting the developing fetus (28). Moreover, BC has been shown to activate inflammatory pathways, and exposure to particulate matter containing BC during pregnancy was associated with increased levels of inflammatory markers in the blood of pregnant women (29). From a genetic perspective, recent studies have unraveled that maternal exposure to traffic-related air pollution, including BC, interacted with certain genetic variants in the mesenchymal epithelial transition (MET) gene, increasing ASD risk (30). The MET gene plays a role in neural development and has been implicated in ASD susceptibility. Another study examined gene–environment interactions between BC exposure and the serotonin transporter gene SLC6A4, and revealed that BC exposure during pregnancy was associated with a higher risk of ASD in children carrying specific SLC6A4 variants (31). These findings highlight the importance of considering individual genetic variations when assessing the impact of BC exposure on ASD risk.

This study has revealed the trends in the OR between monthly mean pregnancy PM_2.5_ exposure and ASD. A key finding was the correlation strength between monthly exposure to PM_2.5_ and ASD during pregnancy was characterized by an approximately right-skewed distribution. This pattern may indicate that the distribution of actual PM_2.5_ concentration absorbed by pregnant women also follows a right-skewed distribution, in which most of the values are concentrated at the lower end of the range, with a smaller proportion of higher values. This skewed distribution suggests that actual PM_2.5_ uptake is likely to be elevated at particular times during pregnancy and may have implications for ASD development. Physiological changes during pregnancy play a role in altering the distribution pattern of PM_2.5_ uptake. Studies have found that during the second trimester of pregnancy, the maternal respiratory system undergoes anatomical and physiological changes, leading to increased tidal volume and respiratory rate (32). Additionally, increased cardiac output during this period enhances pulmonary blood flow (33). These physiological changes may increase the deposition and retention of PM_2.5_ in the respiratory system, leading to higher levels of PM_2.5_ uptake during this period. In the present study, analyses for PM_2.5_ and its components consistently showed the strongest association between PM_2.5_ exposure and ASD risk from month 4 to month 7 of pregnancy, consistent with the findings reported by Guxens and Jung (25). The increase in exposure levels during this period coincided with an important period of fetal development, including organ formation and rapid brain growth. This temporal pattern implies that the second trimester of pregnancy is a critical period during which elevated PM_2.5_ exposure may have a greater impact on fetal brain development, leading to an increased ASD risk.

This study has several limitations. Firstly, since this study was conducted within a psychiatric unit, children were chosen without ASD but with mild symptoms of tics as control group, and although tics are fundamentally different from ASD, their presence as a neurological abnormality may affect the results. Secondly, due to the time constraints of the clinic visit, covariates were not comprehensively assessed, and many social, dietary, lifestyle, and parental-related factors were not included. Furthermore, due to the limitation of the volume of patients attending the clinic, this study did not follow the optimal design of selecting a gender-age matched control group; only gender was matched. Finally, more standardized studies and more sophisticated statistical analyses are needed to delve deeper into the effects of air pollution on ASD.

## 5 Conclusion

Maternal exposure to PM_2.5_ and its components was significantly associated with elevated risk of childhood ASD, with particularly strong associations observed for ${\mathrm{SO}}_{4}^{2-}$ and BC. The period from the 4th to 7th month of gestation was identified as a critical window of vulnerability. As these are observational findings, replication in larger, multi-center cohorts is required to confirm generalizability. Meanwhile, this study highlights the need for targeted public health strategies to mitigate prenatal air pollution exposure and provides important implications for subsequent mechanistic investigations.

## Data Availability

The raw data supporting the conclusions of this article will be made available by the authors, without undue reservation.
